# Supplementary material for: Spatial Variation in Stream-Water Bacterial Communities Across Sites Differing in Wild Chinese Giant Salamander Detection Frequency
Source: Biology (Basel). 2026 Sep 18;15(18):1647. doi: 10.3390/biology15181647 (PMC13604818; doi:10.3390/biology15181647)
Supplement: Supplementary file 1 [file biology-15-01647-s001.zip › Data Related to “Spatial Variation in Stream-Water Bacterial Communities across Sites Differing in Wild Chinese Giant Salamander Detection Frequency”/Microbial Data/result/08.FunctionPrediction/picrust2/heatmap/sample/heatmap.sample.COG.cluster.pdf]

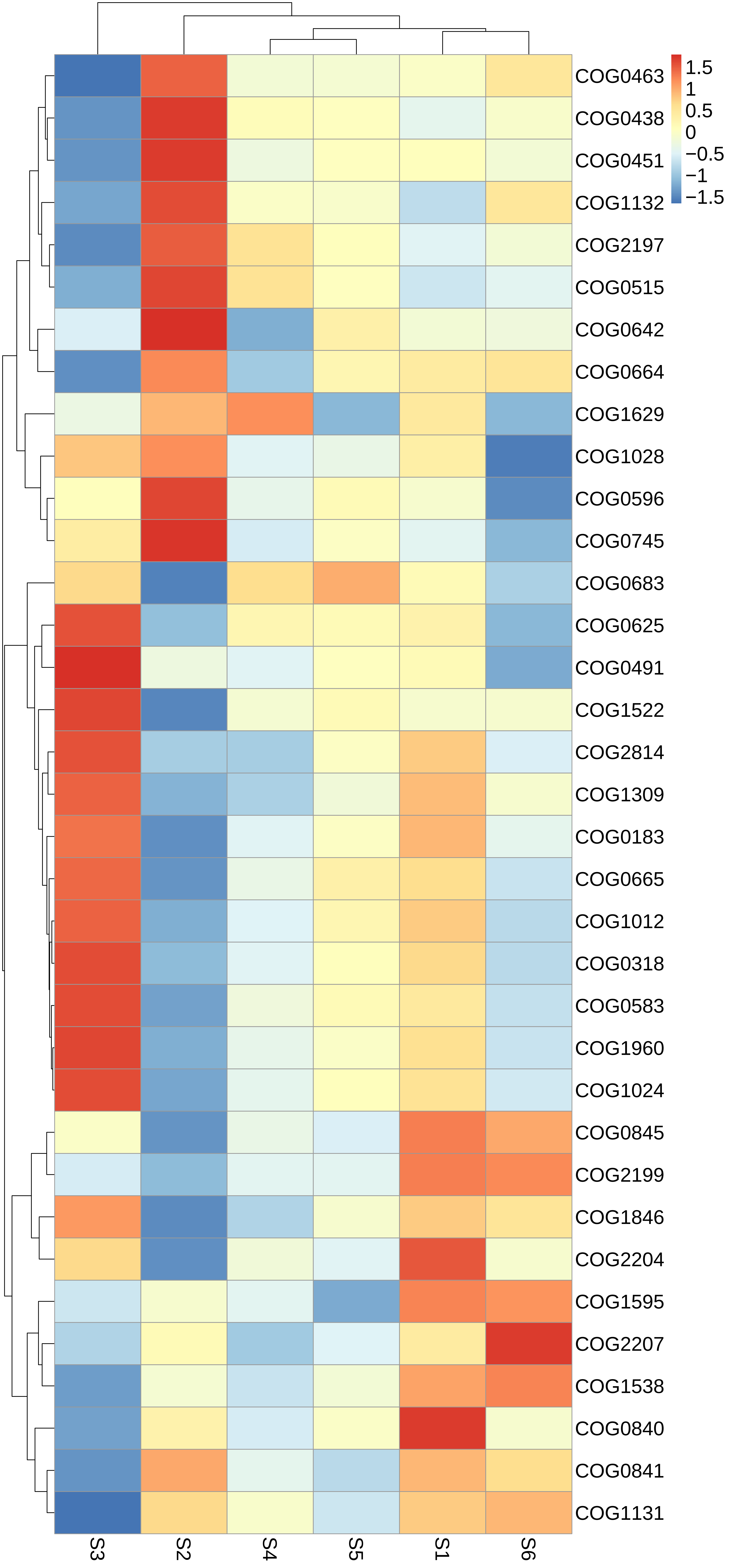

1.5
1
0.5
0
−0.5
−1
−1.5
COG0463
COG0438
COG0451
COG1132
COG2197
COG0515
COG0642
COG0664
COG1629
COG1028
COG0596
COG0745
COG0683
COG0625
COG0491
COG1522
COG2814
COG1309
COG0183
COG0665
COG1012
COG0318
COG0583
COG1960
COG1024
COG0845
COG2199
COG1846
COG2204
COG1595
COG2207
COG1538
COG0840
COG0841
COG1131
S3
S2
S4
S5
S1
S6
